# Acute Cognitively Engaging Exergame-Based Physical Activity Enhances Executive Functions in Adolescents

**DOI:** 10.1371/journal.pone.0167501

**Published:** 2016-12-28

**Authors:** Valentin Benzing, Theda Heinks, Noëmi Eggenberger, Mirko Schmidt

**Affiliations:** 1 Institute of Sport Science, University of Bern, Bern, Switzerland; 2 Department of Pediatric Neurology, Development and Rehabilitation, University Children’s Hospital, Bern, Switzerland; University of Granada, SPAIN

## Abstract

The study aimed to elucidate the influence of cognitive engagement comprised in an acute bout of exergame-based physical activity on executive functions (inhibition, cognitive flexibility) in adolescents. Therefore, the level of cognitive engagement and the intensity of physical activity were systematically varied across three experimental conditions. Sixty-five healthy male adolescents (13–16 years) were randomly assigned to one of three conditions: (a) physical activity with high levels of cognitive engagement during active video gaming, (b) physical activity with low levels of cognitive engagement during active video gaming, (c) sedentary with low levels of cognitive engagement during passive video watching. Manipulation checks, including subjective and objective operationalizations of cognitive engagement, were applied. Executive functions were assessed before and after each condition using the D-KEFS design fluency test. Results showed that cognitive engagement, operationalized by subjects’ ratings and heart rate variability, differed between conditions. The physical activity condition with a high level of cognitive engagement resulted in significantly better performance in cognitive flexibility compared to conditions with low levels of cognitive engagement. Regarding benefits for executive functions in male adolescents, the results indicate that acute physical activity with high cognitive engagement could be more efficient than physical activity of the same intensity with low cognitive engagement. Even though further evidence is needed, these results extend previous research and suggest a methodological approach for measuring cognitive engagement.

## Introduction

A wide range of literature demonstrates the beneficial effects of acute physical activity (PA) on cognitive performance in children and adolescents [1,2], with executive functions profiting most [3]. Since not all types of PA benefit cognition equally [4], PA characteristics, such as intensity, duration and modality remain to be explored [1,4,5]. Recently an increased interest in modality of PA and in specific factors such as cognitive engagement has emerged [4,6]. Cognitive engagement, defined as the level to which the allocation of attentional resources and cognitive effort is needed to master difficult skills [4], seems to be of central importance, as it is supposed to be linked to the effects on executive functions [4,6–8]. However, the empirical evidence concerning the beneficial effects of cognitive engagement in PA on executive function in adolescence is contradictory and limited. Thus the question remains whether and how the level of cognitive engagement inherent in PA might influence executive functions.

Intact executive functions are known to be essential for mental and physical health, academic achievement, everyday life and development [9]. The umbrella term “executive functions” refers to higher-level cognitive functions that manage other more basic functions and enable controlled, goal-directed behavior. It is generally agreed that there are three core executive functions: a) inhibition–involving response inhibition, to control one’s thoughts, attention, emotions and behavior; b) updating–holding information in the working memory and apply it to a task at hand; and c) cognitive flexibility–switching between tasks, changing perspectives, being able to react to changes in environmental demands. Within the core executive functions, cognitive flexibility seems to be the most complex, building upon inhibition and updating [9]. From a developmental perspective, it is the last one to be fully developed and seems to predict academic achievement (e.g., [10]).

Bearing in mind the positive relationship between physical activity and cognition in adolescents (for review see [11]) and the predictive associations of PA during adolescence for cognitive performance in young adults [12], one might be surprised by the small number of studies investigating the effects of single bouts of PA on the different executive function domains in this age group. A recent meta-analysis [2] showed a significant effect of acute PA on executive functions, however when only studies with adolescents are considered, the results of this meta-analysis are limited to three studies exclusively investigating effects on inhibition [2]. No study with adolescents reported effects on updating, whereas two studies investigated cognitive flexibility, one revealing positive effects on cognitive performance [13] and one showing no effects [14]. These contradictory results are difficult to interpret, as the studies differed not only regarding their measures of cognitive performance and quantitative (intensity, duration) but also qualitative PA characteristics (modality).

In the literature, increasing interest in qualitative PA characteristics [15] such as cognitive engagement has emerged [6] as it is proposed to critically influence benefits for executive functions [4]. This assumption is based on the “cognitive stimulation hypothesis”, which suggests that cognitively engaging activities pre-activate the same brain regions that are used to control higher-order cognitive processes, which in turn are needed for performing tasks measuring executive functions [6,16,17]. It further implies that higher cognitive engagement comprised in PA might be associated with better cognitive performance afterwards. However, considering studies investigating the influence of cognitive engagement inherent in PA on the core executive functions, no study has revealed effects on cognitive flexibility. Concerning updating or inhibition (for an overview see, S1 Table), some have revealed benefits [7,18–20], some no influence for the entire study sample [8,21] and some have even found detrimental effects [22,23]. Given these conflicting results and considering the vast procedural differences between studies [4], drawing ultimate conclusions regarding the influence of cognitive engagement inherent in PA on executive functions is nearly impossible.

Procedural differences in study designs might impede existing research about the effects of modality on cognitive performance. Previous studies recommend systematically modifying the amount of cognitive engagement comprised in PA [21], whereby in most studies two completely different activities were investigated [7,18]. This seems problematic since possible confounders such as social interactions are not controlled and it is therefore difficult to attribute benefits in cognition to either the physical or the cognitive parameters inherent in the respective activity [6]. Therefore, research designs are needed using standardized PA, only modifying the degree of cognitive engagement without changing the amount of social interactions, under controlled conditions within the field.

One research tool to combine the advantages of highly controlled PA within the field is exergaming, a portmanteau of “exercise” and “gaming” [24]. Exergaming is active video gaming that has been proven to be physically challenging [25] and motivating [26]. It enables researchers to manipulate PA characteristics under controlled circumstances. So far, exergaming has been used in one study investigating the effects of cognitive engagement inherent in acute PA on executive functions. Results show that children’s performance on tasks measuring executive functions was found to be improved immediately after the intervention [8]. In that study, exergaming was proven to be feasible, highly standardized and able to modulate activity characteristics specifically. However, the question of whether the degree of cognitive engagement inherent in the exergames affected cognitive outcomes remains unanswered: on the one hand, both experimental conditions might have been cognitively engaging, on the other hand cognitive engagement itself was not operationalized.

In general, research is lacking a reliable and sensitive assessment of the level of cognitive engagement during PA to clarify the potential relationship between PA, cognitive engagement and cognitive outcomes [4]. To date, only few studies examining cognitive engagement comprised in PA have tried to measure the cognitive challenges of specific PA. Mainly observational data [27,28] or a more general measure of engagement in the activity [8] has been used. However, a subjective rating on the individual level seems to be important in order to consider cognitive overload as a confounding variable. Besides these approaches, in other areas of research, early attempts have tried to measure cognitive engagement using psychophysiological methods such as heart rate [29] and more recently heart rate variability (HRV) [30]. HRV is an important marker of the autonomic nervous system, reflecting the interplay between the sympathetic nervous system and the parasympathetic nervous system [31]. Since the association of HRV with cognitive performance is strengthened after PA [31] and a faster vagal recovery after cognitive engagement seems to be associated with better performance in cognitive flexibility [32], one would therefore assume that the level of cognitive engagement during PA affects subsequent performance and HRV. Based on the cognitive stimulation hypothesis, a higher cognitive demand during PA for the same intensity of PA should then be associated with faster vagal recovery (higher HRV) after the experimental manipulation (and vice versa).

In summary, acute PA seems to promote executive functions [1,2], which are of major importance for health and academic achievement [9]. Cognitive engagement in PA is proposed as one important factor fostering these benefits, although the operationalization of cognitive engagement is insufficient so far and studies investigating this relationship might be impeded by procedural differences [4]. In addition, there is a lack of research on adolescence, the reported results are contradictory [13,14] and only a single study specifically investigated the influence of cognitive engagement in PA [7]. The aim of the present study was therefore to clarify the impact of cognitive engagement comprised in PA on executive functions in male adolescents. A randomized controlled trial was conducted comparing the cognitive performance of three different groups (two PA groups differing in the level of cognitive engagement and one control group). Cognitive engagement was specifically addressed using ratings of perceived cognitive engagement and HRV. With regard to procedural differences and standardization, the current study used exergaming to realize the experimental conditions. In addition, measures of potential confounding variables (pleasure, arousal, enjoyment of activity) were applied. Our hypotheses were that an acute bout of PA would enhance executive functions depending on the degree of cognitive engagement. Furthermore, we expected the measures of cognitive engagement to sensitively differentiate between the high and low cognitive engagement conditions.

## Materials and methods

### Subjects

Sixty-five male adolescents (grade 7–9) between the ages of 13 and 16 years (*M* = 14.51, *SD* = 1.08), recruited from two secondary schools in Bern, Switzerland participated in the study. Of the 69 participants initially invited, four were unavailable due to sick leave or injuries. Only male adolescents were invited since prior experience with exergaming/video gaming and HRV measures vary between the two sexes (e.g. [33,34]). The Institutional Review Board of the Faculty of Human Sciences at the University of Bern approved the study protocol, which adhered to the latest version of the declaration of Helsinki. The legal guardians of all subjects provided informed written consent and adolescents agreed to participate. The exclusion criteria were any neurological, developmental, or medical condition that would affect the subjects’ integrity or study results. Loss of data was evident in five cases (7.7%), due to problems with the heart rate (HR) belt. Since Little’s “missing completely at random test” was not significant (χ^2^ (89) = 82.00, *p* = .688), the resulting missing values were imputed with the help of the expectation-maximization (EM) algorithm. The pattern of results did not change with or without imputation and the imputed data was used for all the analyses. Considering previous studies [35,36] investigating acute effects of exergaming activities on executive functions using the design fluency test, an a priori power analysis [37] with (1 –beta error probability = .80; alpha error probability = .05; effect size *f* = .15; number of groups = 3, number of measurements = 2; and correlation between the repeated measures *r* = .75) was performed. This resulted in a minimal sample size of 57 subjects. The background variables did not differ between groups (see Table 1).

**Table 1:** Means, standard deviations and test statistics comparing background variables between groups.

	Shape Up (*n* = 21)	Running (*n* = 23)	Control (*n* = 21)		
	*M* (*SD*)	*M* (*SD*)	*M* (*SD*)	*F*(2,61)	*P*
Age (years)	14.52 (1.03)	14.61 (1.22)	14.38 (1.02)	0.24	.785
Body mass index (kg/ m^2^)	20.21 (2.24)	20.35 (1.87)	20.56 (2.80)	0.12	.889
Socioeconomic status	7.06 (1.73)	6.72 (1.45)	7.24 (1.52)	0.60	.550
Pubertal status	7.58 (1.95)	7.51 (1.98)	7.52 (2.37)	0.01	.994
Physical activity behavior (minutes/week)	258.71 (260.03)	226.77 (161.81)	225.03 (185.41)	0.18	.835

### Design

In a between-subjects design, participants were randomly assigned to one of three conditions: (a) *Shape Up* group (PA with high levels of cognitive engagement); (b) *Running* group (PA with a low level of cognitive engagement); and (c) *Control* group (sedentary with a low level of cognitive engagement). Based on the theoretical assumption of underlying shared information processes in motor and cognitive control, for condition (a) a non-automated activity was chosen with high demands on coordination, which might pre-activate the same brain regions that are used to control higher order cognitive processes [7]. In contrast, for condition (b) a highly automated, physically exerting, but not cognitively engaging activity was set. Since the goal of the study was to address the practical question of what type of PA should be used to improve executive functions, two PAs which differ in the level of cognitive engagement were compared to a control group.

For each condition, the same tests were completed and conducted at the same time in three unused, quiet classrooms of the respective schools. Experimental conditions were conducted using the XBOX Kinect (Microsoft, Redmond, WA). This is a game console combined with a motion-sensing input device. Users are able to control and interact with the console through their body movements. The integrated cameras enable the user to be projected directly onto the screen into the virtual reality.

### Procedure

Informed consent was obtained and subjects were numbered previous to testing; these numbers were randomly assigned to one of three groups (3 sets; 23 numbers each) using randomizer.org. On the day of testing, data on background variables were gathered–date of birth, height, weight, pubertal status [38], socioeconomic status [39] and PA behavior [40]. Subjects were asked not to engage in any PA before the assessment; teachers ensured that subjects did not participate in physical education before. They were fitted with HR-monitoring equipment and assigned to their respective conditions. Executive functions performance was measured before (pre-test) and after (post-test) the activity. Each condition lasted about 15 minutes including two short breaks of approximately 20 seconds. During the second break, subjects filled out pleasure and arousal ratings. Immediately after the activity (post-test), perceived physical exertion, cognitive engagement and enjoyment of the activity were measured. At the end, the HR monitoring equipment was removed. Subjects received a small gift and were dismissed back to class.

### Conditions

In the *Shape Up* condition, both the self-determined intensity of the PA and cognitive engagement were high. Subjects played three different “workouts” of the “Shape Up” game (Ubisoft, Montreal, Canada), in which they had to imitate and learn new sequences of movements, comparable to aerobics. A higher score can be achieved with more movement and more accurate performance of the movement sequences.

In the *Running* condition, the self-determined intensity of the PA was high while the level of cognitive engagement was low. Thus, subjects played “run the world” included in the “Your Shape^TM^ Fitness Evolved 2012” (Ubisoft, Montreal, Canada) game. In this game, subjects have to run through virtual streets and engage in speed and knee up challenges. A higher score can be achieved by running faster.

In the *Control* condition, in order to sustain low levels of PA intensity and low levels of cognitive engagement, subjects watched a video on a computer, showing a documentary report about mountain running.

### Background variables

*Pubertal status* was assessed using the German version of the pubertal developmental scale [36]. This consists of three questions for each gender, asking for example: “Have you noticed a deepening of your voice?”. Responses are given on a 4-point Likert scale, scoring 1–4 points (e.g., not yet started; barely started; definitely started; seems complete). The puberty index (3–12 points) is calculated by summing up the scores of the three items. An acceptable reliability and validity has been demonstrated [38]. *Socioeconomic status* [39] was assessed using the Family Affluence Scale II. This consists of four questions asking about the family (whether they have their own bedroom, the number of family-owned cars, computers and holidays in the past year). The response format varies by item and points are given for a higher number, for example of computers. The prosperity index is then calculated as the sum of the points on the four items. An acceptable reliability and validity has been demonstrated [36]. *PA behavior* was assessed using the PA, exercise, and sport questionnaire [40]. Respondents are asked to indicate the frequency and average duration of up to three types of exercise they regularly engage in. PA behavior in minutes per week is then calculated. Acceptable psychometric properties have been demonstrated [40].

### Manipulation check

To test whether experimental manipulation had succeeded, subjective and objective measures were used. The rating of perceived physical exertion (RPE) was used as a subjective measure of *physical exertion* [41]. The HR was recorded using the Polar Team^2^ Pro system (Polar Electro Oy, Kempele, Finland) as an objective measure. As a subjective measure of *cognitive engagement* (rating of perceived cognitive engagement; RCE), the RPE scale was adapted to specifically ask about the perceived cognitive engagement of the activity. Even though it has not been validated, this instrument has been proven to be feasible in a study with primary school children [42]. As our objective measurement, we used time domain measures of the HRV, because they seem to be most sensitive to cognitive engagement [30]. Since the HRV narrows during PA, differences between experimental groups during the intervention are improbable. Therefore, we considered the HRV during the recovery period (post-test) after the PA to be the most sensitive measure of the triggered effects of cognitive engagement during PA (pre-test HRV: –5 to 0 minutes before the activity started; HRV during: 15 minutes during activity; post-test HRV: 0 to 5 minutes after the PA ended). Additionally, *control variables*, such as the enjoyment of the activities and the rated pleasure and arousal using the Self-Assessment-Manikin [43] were measured. The enjoyment was measured using three questions: 1) “How much did you like the activity?” 2) “Did you feel comfortable doing the activity?” 3) “Did you enjoy doing the activity?”. The questions had to be answered on a three-item 4-point Likert scale (0–3 points for each item) and the sum was calculated as the enjoyment score. The Self-Assessment Manikin is a widely used non-verbal pictorial assessment technique to measure a person’s affective reaction to a variety of stimuli. It consists of one item for each construct. Acceptable psychometric properties have been demonstrated [39].

### Executive Functions

Executive functions were measured using the design fluency test from the Delis-Kaplan Executive Function System [44]. This system is one of the most widely used test batteries for assessing executive functions [45], with retest reliabilities ranging from .62 to .80 [46]. Sensitivity to distinguish clinical from control groups has been shown [46]. The design fluency test has also been used in previous studies on active video gaming and executive functions [32,33]. It seems to require higher executive function skills and underlying component skills [44] and it is believed that, on a fundamental component skills level, it measures visual attention, motor speed, visual perceptual skills and constructional skills. In terms of executive functions, it seems to target the initiation of problem-solving behavior, fluency, creativity, simultaneous processing, inhibition, and cognitive flexibility [44]. The task contains three trials, each consisting of 35 boxes with a series of five dot matrices inside. In every box, five dots have to be connected using four lines. Subjects have 60 seconds to create as many novel designs as possible. In the first trial, the boxes contain only solid dots that can be connected. In the next two trials, there are five solid and five empty dots inside each box. During the second trial, subjects are instructed to connect only empty dots; in the third trial, subjects have to alternate between connecting a solid and an empty dot. In detail, the first trial (fluency trial) measures design fluency (generation of novel designs), the second trial (inhibition trial) additionally measures response inhibition, and the third trial (cognitive flexibility trial) measures the generation of novel designs while switching. The resulting raw scores were transformed to scaled scores [44].

### HRV analyses

In accordance with recommended standards of measurement, HRV was used to assess autonomic nervous system function [47]. HR was recorded using the Polar Team^2^ Pro system. Cardiac interbeat (RR) intervals were exported using the Polar Team^2^ Pro software, preprocessed and analyzed [48] using Kubios (Biosignal Analysis and Medical Imaging Group, University of Eastern Finland, Joensuu, Finland). Data was visually inspected and artifact correction was applied to exclude RR intervals which differed from the previous and subsequent interval by more than 15%. Removed RR intervals were replaced using cubic spline interpolation. The smoothness prior method with a lambda value of 500 was applied to remove affecting trend components. The root mean square of successive differences (RMSSD) was calculated as a measure of HRV because its statistical properties are preferred over other time domain measures [47].

### Statistical analyses

Statistical tests were performed using SPSS 23.0 (SPSS Inc., Chicago, IL, USA). Preliminary analyses were calculated using ANOVAs for between-subjects comparisons of background variables, manipulation check variables (physical exertion, cognitive engagement), control variables (pleasure, arousal, and enjoyment ratings) and dependent variables at pre-test. For background and dependent variables at pre-test, no significant differences were observed (*P*s > .05). For significant differences in manipulation check variables, LSD post-hoc comparisons were used to determine the specific differences between the three groups. To compare executive function performance, an ANCOVA was performed using pre-test values as covariates and post-test values as dependent variables [49,50]. LSD post-hoc comparisons were used to determine differences between specific groups. To examine potential differential effects, an additional ANCOVA was conducted including all background variables as covariates (age, BMI, pubertal status, socioeconomic status, PA behavior). The level of significance was set at *P* < .05 for all analyses.

## Results

### Manipulation check

The manipulation checks revealed significant group effects with regard to physical exertion and cognitive engagement (see Table 2). Post-hoc analyses examined overall differences in further detail: as intended, *physical exertion* was increased in Running and Shape Up compared to Control (RPE: *P* < .0005; HR: *P* < .0005), but did not differ between the two PA groups (RPE: *P* = .111; HR: *P* = .142). Considering *cognitive engagement*, the Shape Up activity was perceived to require more effort than Running (*P* = .022) and Control (*P* = .001), but Running and Control did not differ (*P* = .201). The post-intervention RMSSD differed between the three groups (Running vs. Shape Up: *P* = .022; Control vs. Running: *P* < .0005; Control vs. Shape Up: *P* < .0005). Concerning the control variables, a significant group effect was obtained only in arousal ratings. Not surprisingly, the level of arousal was increased in Shape Up (*P* = .002) and Running (*P* = .001) compared to Control, but did not differ between the two PA groups (*P* = .835).

**Table 2:** Descriptive statistics and between-group analyses of manipulation check and control variables.

	Shape Up (*n* = 21)	Running (*n* = 23)	Control (*n* = 21)			
			
	*M* (*SD*)	*M* (*SD*)	*M* (*SD*)	*F*(2,61)	*P*	η ^2^_p_
*Physical exertion*						
RPE	14.76c (1.84)	13.65c (2.44)	8.00a,b (2.47)	53.76	< .0005	.634
HR pre	86.93 (10.12)	86.21 (15.36)	80.59 (10.12)	1.66	.199	.051
HR during	141.54c (23.25)	152.11c (27.13)	82.39a,b (19.20)	54.73	< .0005	.638
HR post	100.44c (12.77)	105.93c (15.04)	79.44a,b (8.47)	26.22	< .0005	.463
*Cognitive engagement*						
RCE	12.71b,c (2.55)	10.87a (2.69)	9.86a (2.54)	6.56	.003	.175
RMSSD pre	44.58 (16.20)	42.48 (19.93)	46.27 (14.61)	0.27	.765	.009
RMSSD during	14.86c (9.06)	15.42c (9.49)	47.44a,b (17.25)	47.99	< .0005	.608
RMSSD post	25.99b,c (18.33)	16.67a,c (9.85)	47.35a,b (16.02)	30.89	< .0005	.499
*Control variables*						
Pleasure	6.81 (1.44)	7.04 (1.19)	7.52 (0.98)	1.87	.159	.058
Arousal	6.19c (1.47)	6.30c (1.82)	4.38a,b (2.06)	7.77	.001	.199
Enjoyment	6.57 (1.47)	6.39 (2.10)	6.67 (1.93)	0.13	.883	.004

Note. RPE: Rating of Perceived Exertion. HR: Heart Rate. RCE: Rating of Cognitive Engagement. RMSSD: Root Mean Square of Successive Differences. Significant differences of post-hoc (LSD) comparisons are indicated by respective letters (a = Shape Up; b = Running; c = Control).

The results indicate that whereas intensity of PA was increased equally in the experimental groups compared to the Control group, cognitive engagement was elevated only in the Shape Up group. These results are supported by both subjective and objective data.

### Executive functions

Regarding executive function performance (see Table 3), a significant group effect was obtained in the cognitive flexibility trial of the design fluency test (*F* (2, 61) = 3.50, *P* = .036, η ^2^_p_ = .103). Post-hoc comparisons revealed that in this trial, the Shape Up group performed significantly better than the Control group (*P* = .040) and the Running group (*P* = .017); the Running and Control groups did not differ concerning their performance (*P* = .770). In the fluency and inhibition trials, no significant group effects (*P*s > .05) were observed. These results indicate that solely the Shape Up group was able to improve its performance in cognitive flexibility after the activity.

**Table 3:** Executive function performance in D-KEFS design fluency test of the experimental conditions.

	Shape Up (*n* = 21)	Running (*n* = 23)	Control (*n* = 21)
	*M* (*SD*)	*M* (*SD*)	*M* (*SD*)
*Design fluency pre-test*			
Fluency	10.76 (2.32)	11.04 (2.65)	10.71 (2.49)
Inhibition	10.62 (3.09)	11.57 (2.69)	12.14 (3.00)
Cognitive flexibility	11.86 (3.12)	12.43 (2.83)	11.29 (3.12)
*Design fluency post-test*			
Fluency	13.38 (2.62)	13.96 (3.18)	13.81 (2.94)
Inhibition	13.62 (2.69)	13.52 (2.59)	13.29 (3.51)
Cognitive flexibility	14.05b,c (2.90)	12.52a (2.94)	12.19a (2.62)

Note. Significant differences of post-hoc comparisons (LSD) are indicated by the respective letters (a = Shape Up; b = Running; c = Control).

An additional ANCOVA was conducted to examine potential differential effects. The pattern of results did not change after additionally controlling for all background variables (*F* (2, 56) = 3.75, *P* = .030, η ^2^_p_ = .118). No significant effects in the fluency and inhibition trials were observed (*P*s > .05). However, none of the additionally included covariates had a significant effect (*P*s > .05), indicating no differential effects of the interventions investigated.

## Discussion

The aim of the study was to investigate the effects of cognitive engagement inherent in PA on executive functions in male adolescents. First, cognitively engaging PA outperformed simple aerobic activity on a test of cognitive flexibility. Second, the subjective (rating of cognitive engagement) and objective measures (HRV at post) of cognitive engagement differed between the respective experimental conditions.

With respect to prior studies investigating the effects of cognitive engagement in acute PA on executive functions in children and adolescents, a uniform interpretation is complicated because of procedural differences between the studies. Concerning the assessment of executive functions, most previous studies considered inhibition as a dependent variable. Only one study examined the effects on all three core executive functions (inhibition, updating, cognitive flexibility) [19], finding no beneficial effects of cognitively engaging PA. These findings contradict the results of the current study, which included an older study sample. Therefore, the conflicting results might be explained by the developmental trajectory of the executive functions. Inhibition is the first executive function to be fully developed, cognitive flexibility the latest [9]. Adolescents might benefit as cognitive flexibility seems to be prone to positive changes of PA throughout later stages of child development. Therefore, one might speculate that aspects of executive functions which are not yet fully developed (cognitive flexibility) might be easier to change [27]. The relationship between age and the responsiveness to acute PA in terms of benefits on executive functions needs to be explored in future studies.

Concerning modality, in contrast to most of the studies conducted so far [7,18,22,23], the current study varied the level of cognitive engagement systematically using an individual level and standardized activities (exergaming) in order to prevent possible confounds. It becomes clear that when comparing circuit training with team games [18], for example, both conditions might differ in various variables such as the amount of social interaction, cognitive engagement or the physical demands of the task, which might individually or together affect executive function differentially [7,8,18]. By using an additional third condition, the findings of the current study extend existing knowledge, showing that pure aerobic exercise was not able to improve executive functions compared with a non-exerting control condition. However, a major limitation of the current study is that a sedentary cognitively engaging condition is missing. Because the main aim of the study was to investigate a question of practical relevance–whether pure aerobic or cognitively engaging exergaming is superior in improving executive functions–an additional fourth condition was not applied. Consequently, one cannot determine whether a sedentary cognitively engaging condition would have revealed the same effects, or whether a combination of PA and cognitive engagement is crucial in order to improve executive functions. To systematically examine the single and/or combined effects of cognitive engagement and PA on executive functions, more controlled studies are needed in individual settings using similar physical task demands and differing only in the level of cognitive engagement, using a 2 × 2 experimental design, so as to explore the underlying mechanisms [8,19].

Concerning intensity and duration, the current study used an activity of 15 minutes at a moderate to vigorous intensity. Duration and intensity varied extensively in previous studies investigating cognitive engagement (for an overview, see S1 Table), although a tendency favoring shorter activities can be observed. Detrimental effects were only found in the study with the longest duration (50 minutes) at moderate to vigorous intensity [22,23]. Therefore, in the study of Gallotta et al. [22,23] duration might have been too long to foster immediate benefits for cognition. Chang et al. [51] recently compared PA lasting 10, 20 and 45 minutes, recommending 20 minutes PA exerting the most benefits on cognition. Since other recommendations range between 11–20 minutes at moderate to vigorous intensity [52] and the study of Chang et al. was conducted with adults [51], 15 minutes might be a reasonable time frame for adolescents. Because recommendations mostly evolve from investigations with adults, it is unclear whether the same mechanisms apply for adolescents. Future studies should tackle this limitation by investigating the dose-response relation in children and adolescents.

Concerning the age of the study population, adolescence constitutes a critical period of life for the maturing process of cognition and is therefore a predictor of adult health [53]. However, studies investigating adolescents are scarce. In keeping with the current study, it is noticeable that especially those studies conducted with older children/ adolescents [7,18] seem to have found more benefits for cognition than studies conducted with younger children [22,23]. Although these findings have to be interpreted cautiously, due to the empirical evidence being in general limited, nonetheless, both PA and cognitive engagement as well as their effects on cognition might be differentially influenced by age and child development. Therefore, age and additional related aspects, such as the developmental status of the subjects, might be important issues for future studies.

With regard to gender, only male adolescents were included in the current study. The reasons for this were twofold. First, the degree of prior experience with exergaming and video gaming varies between the two genders in youth [33]. Second, existing gender differences in heart rate variability in adolescents might influence HRV analyses [34,54]. This means that any generalization of the study’s results is limited to male adolescents. Future studies should therefore investigate whether exergaming could also be a viable tool to promote positive effects on executive functions in female subjects.

Regarding highly standardized activities to investigate cognitive engagement, empirical evidence is limited. So far, only one study has considered exergaming as a tool for comparing PA differing in levels of cognitive demand [8]. However, according to the author, the role of cognitive engagement in PA could not be fully determined, as the experimental group might not have differed in the level of cognitive engagement. This highlights that an operationalization differentiating between the high and low cognitive engagement conditions can be seen as a minimum requirement in order to attribute increased cognitive performance to cognitive engagement comprised in PA. One limitation of the current study is that other important variables (besides cognitive engagement) might be responsible for alterations in cognitive performance. Physical as well as cognitive stress could serve as an alternative explanation [55] and might be associated with executive functions [20,56] and manual motor performance [57]. Therefore, in addition to cognitive engagement, future studies could measure stress induction using an objective indicator, such as cortisol.

Compared to the existing body of research, this is the first study to propose a subjective and objective operationalization of cognitive engagement in acute PA. For the subjective ratings, the question arises whether adolescents are able to correctly estimate cognitive engagement inherent in PA–based on the current study and one study in younger children [42], we suppose that this question could be answered in the affirmative. However, as the rating scale used has not yet been validated, one cannot be sure whether it really reflects cognitive engagement. For the objective assessment after the activity, one can only speculate whether HRV reflects cognitive engagement during PA [30]. It also seems possible that it reflects the amount of cognitive engagement during the cognitive task [58] or task performance itself [30,32]. Due to the reciprocal associations of cognitive engagement and cognitive performance, it remains unclear which psychological construct might be reflected by physiological processes. Since HRV differed significantly between the two experimental conditions whereas HR did not, we assume that the different PA characteristics, including the level of cognitive engagement, did indeed impact HRV. Nevertheless, the results and the validity of the objective and subjective measures need to be confirmed in future studies.

The cognitively engaging PA of the current study can be regarded as rather high in cognitive demand, as supported by subjective ratings. Although, depending on their cognitive abilities, some subjects might have been cognitively under- or overloaded, resulting in a lowered cognitive performance after PA. This might have influenced the results, as neither the amount of cognitive demand, nor the intensity of the PA was adjusted on an individual level in the current study. We therefore cannot be sure that all individuals were challenged with an optimal level of intensity and/or cognitive demand. This might have resulted in an underestimation of the potential benefits for cognitive performance. Future studies should search for the optimal challenge point in cognitive engagement and adjust both intensity of PA and cognitive engagement on an individual level [28, 52].

Regarding potential differential effects, an existing heterogeneity in certain sample characteristics, such as environmental (socioeconomic status), developmental (age, pubertal status) or physical (physical fitness, BMI) factors, raises the question whether all adolescents benefit equally from acute exergaming. The existing literature seems to support differential effects of acute PA on cognitive performance (e.g. [52]) indicating, for example, that participants with a higher level of fitness might profit more from acute PA in terms of cognitive performance (e.g. [21, 59]). The results of the analyses examining differential effects of the current study therefore seem contradictory to the literature on adolescents [59], because no moderating variables were discovered. This would indicate that the benefits of acute exergaming are independent of participants having specific characteristics. However, the study population was deliberately designed to be homogenous, which results in a smaller variance, reducing potential differential effects. In addition, the current study was not very well suited to detecting differential effects. Therefore, future studies could examine the potential influence of specific participant characteristics on executive functions in exergaming.

Considering the existing literature [52, 60], we expected both PAs to have positive effects on executive functions. Surprisingly, subjects in the Running condition did not perform better than controls regarding cognitive flexibility. This finding seems to contradict studies on acute effects of aerobic PA in children [61]. However, focusing on adolescents, only one study has so far observed positive effects on cognitive flexibility [13]. In contrast to this and other studies [14], Berse et al. [13] used interval training of a rather high intensity (10–14 minutes). Therefore, intensity might have been too low in the current study, which could have been responsible for the diverging results. Since according to Hillman et al. [60] acute exercise effects rely on the interplay of severeal factors, such as age, duration, intensity, modality and cognitive requirements of the task, the findings of the current study–positive effects on cognition in the Shape Up but not in the Running condition–emphasize the importance of modality. This is in line with Budde et al. [7], demonstrating that coordinative exercise benefits cognitive performance compared with pure aerobic exercises. They explained their findings in terms of a pre-activation of parts of the brain which are also responsible for mediating executive functions (e.g. attention). In sum, this indicates that a cognitively stimulating exercise might be advantageous in promoting benefits on executive functions. Future studies should examine the interplay and magnitude of factors influencing effects on executive functions (e.g., age, duration, intensity and modality) in acute physical activity.

Referring to previous research which finds the most consistent effects of physical exercise on inhibition [3,52], the results of the current study might seem contradictory. However, the finding that performance in inhibition (second trial) did not improve, might be explained by the different demands of each condition of the design fluency test [62]. In the first trial, productivity/creativity and visual perceptual speed are crucial for the performance. In the second trial, the ability to ignore extraneous stimuli is additionally targeted. However, the first two trials mainly depend on the same abilities–motor planning and motor speed [62]; as a consequence, the inhibition of extraneous stimuli in the second trial is subordinate to task performance. In contrast, the third trial seems to rely mainly on visual attention, reflecting a different construct, which should be interpreted separately [62]. Nonetheless, a limitation of the study is that inhibition was not tested with a classical inhibition task and not all three core domains of executive functions were assessed. Unfortunately, time was limited as testing took place during school hours. Therefore, in future studies, effects on all three core executive functions should be investigated.

## Conclusion

The current study extends existing findings by suggesting a methodological approach for measuring cognitive engagement. Higher order cognition was immediately enhanced by acute, cognitively engaging, exergame-based PA. Thus, our results underline the important role cognitive engagement seems to play in PA in order to foster benefits in cognitive performance. They also suggest that exergaming might serve as a promising tool not only to increase PA levels, but also to enhance executive functions in male adolescents.

## Supporting Information

S1 Table Overview of studies examining acute effects of physical exercises on cognitive performance, differing in the level of cognitive engagement. (DOCX)

S1 Dataset Dataset underlying the findings of the current study. (SAV)

## References

[1] Donnelly JE, Hillman CH, Castelli D, Etnier JL, Lee S, Tomporowski P, et al Physical activity, fitness, cognitive function, and academic achievement in children. Med Sci Sport Exerc. 2016;48: 1197–1222. 10.1249/MSS.0000000000000901 PMC4874515 27182986

[2] Verburgh L, Königs M, Scherder EJA, Oosterlaan J. Physical exercise and executive functions in preadolescent children, adolescents and young adults: a meta-analysis. Br J Sports Med. 2014;48: 973–979. 10.1136/bjsports-2012-091441 23467962

[3] Barenberg J, Berse T, Dutke S. Executive functions in learning processes: Do they benefit from physical activity? Educ Res Rev. 2011;6: 208–222.

[4] Tomporowski PD, McCullick B, Pendleton DM, Pesce C. Exercise and children’s cognition: The role of exercise characteristics and a place for metacognition. J Sport Health Sci. 2015;4: 47–55.

[5] Garber CE, Blissmer B, Deschenes MR, Franklin BA, Lamonte MJ, Lee IM, et al American College of Sports Medicine position stand. Quantity and quality of exercise for developing and maintaining cardiorespiratory, musculoskeletal, and neuromotor fitness in apparently healthy adults: guidance for prescribing exercise. Med Sci Sport Exerc. 2011;43: 1334–1359. 10.1249/MSS.0b013e318213fefb 21694556

[6] Pesce C. Shifting the focus from quantitative to qualitative exercise characteristics in exercise and cognition research. J Sport Exerc Psychol. 2012;34: 766–786. Available: http://www.ncbi.nlm.nih.gov/pubmed/23204358 23204358 10.1123/jsep.34.6.766

[7] Budde H, Voelcker-Rehage C, Pietraßyk-Kendziorra S, Ribeiro P, Tidow G. Acute coordinative exercise improves attentional performance in adolescents. Neurosci Lett. 2008;441: 219–223. 10.1016/j.neulet.2008.06.024 18602754

[8] Best JR. Exergaming immediately enhances children’s executive function. Dev Psychol. 2012;48: 1501–1510. 10.1037/a0026648 22148945

[9] Diamond A. Executive functions. Annu Rev Psychol. 2013;64: 135–168. 10.1146/annurev-psych-113011-143750 23020641 PMC4084861

[10] Yeniad N, Malda M, Mesman J, van Ijzendoorn MH, Pieper S. Shifting ability predicts math and reading performance in children: A meta-analytical study. Learn Individ Differ. 2013;23: 1–9.

[11] Esteban-Cornejo I, Tejero-Gonzalez CM, Sallis JF, Veiga OL. Physical activity and cognition in adolescents: A systematic review. J Sci Med Sport. 2015;18: 534–539. 10.1016/j.jsams.2014.07.007 25108657

[12] Jackson D, Beaver K. The role of adolescent nutrition and physical activity in the prediction of verbal intelligence during early adulthood: a genetically informed analysis of twin pairs. Int J Environ Res Public Health. 2015;12: 385–401. 10.3390/ijerph120100385 25568969 PMC4306868

[13] Berse T, Rolfes K, Barenberg J, Dutke S, Kuhlenbaumer G, Volker K, et al Acute physical exercise improves shifting in adolescents at school: evidence for a dopaminergic contribution. Front Behav Neurosci. 2015;9: 196 10.3389/fnbeh.2015.00196 26283937 PMC4517060

[14] Kubesch S, Walk L, Spitzer M et al A 30-minute physical education program improves students' executive attention. Mind Brain Educ. 2009;3: 235–242.

[15] Esteban-Cornejo I., Gómez-Martínez S., Tejero-González C. M., Castillo R., Lanza-Saiz R., Vicente-Rodríguez G., … Martinez-Gomez D. Characteristics of extracurricular physical activity and cognitive performance in adolescents. The AVENA study. J Sports Sci. 2014;32: 1596–1603. 10.1080/02640414.2014.910607 24779379

[16] Tomporowski PD, Davis CL, Miller PH, Naglieri JA. Exercise and children’s intelligence, cognition, and academic achievement. Educ Psychol Rev. 2008;20: 111–131. 10.1007/s10648-007-9057-0 19777141 PMC2748863

[17] Best JR. Effects of physical activity on children’s executive function: contributions of experimental research on aerobic exercise. Dev Rev. 2010;30: 331–551. 21818169 10.1016/j.dr.2010.08.001 PMC3147174

[18] Pesce C, Crova C, Cereatti L, Casella R, Bellucci M. Physical activity and mental performance in preadolescents: Effects of acute exercise on free-recall memory. Ment Health Phys Act. 2009;2: 16–22.

[19] Jäger K, Schmidt M, Conzelmann A, Roebers CM. Cognitive and physiological effects of an acute physical activity intervention in elementary school children. Front Psychol. 2014;5: 1473 10.3389/fpsyg.2014.01473 25566148 PMC4270126

[20] Schmidt M, Egger F, Conzelmann A. Delayed positive effects of an acute bout of coordinative exercise on children’s attention. Percept Mot Skills. 2015;121(2),431–446. 10.2466/22.06.PMS.121c22x1 26474438

[21] Jäger K, Schmidt M, Conzelmann A, Roebers CM. The effects of qualitatively different acute physical activity interventions in real-world settings on executive functions in preadolescent children. Ment Health Phys Act. 2015;9: 1–9.

[22] Gallotta MC, Emerenziani GP, Franciosi E, Meucci M, Guidetti L, Baldari C. Acute physical activity and delayed attention in primary school students. Scand J Med Sci Sport. 2015;25: e331–8. 10.1111/sms.12310 25134779

[23] Gallotta MC, Guidetti L, Franciosi E, Emerenziani GP, Bonavolonta V, Baldari C. Effects of varying type of exertion on children’s attention capacity. Med Sci Sport Exerc. 2012;44: 550–555. 10.1249/MSS.0b013e3182305552 21814148

[24] Best JR. Exergaming in youth. Z Psychol. 2013;221: 72–78. 10.1027/2151-2604/a000137 25097828 PMC4119754

[25] Barkley JE, Penko A. Physiologic responses, perceived exertion, and hedonics of playing a physical interactive video game relative to a sedentary alternative and treadmill walking in adults. J Exerc Physiol. 2009;12: 12–22.

[26] Penko AL, Barkley JE. Motivation and physiologic responses of playing a physically interactive video game relative to a sedentary alternative in Children. Ann Behav Med. 2010;39: 162–169. 10.1007/s12160-010-9164-x 20169428

[27] Schmidt M, Jäger K, Egger F, Roebers CM, Conzelmann A. Cognitively engaging chronic physical activity, but not aerobic exercise, affects executive functions in primary school children: a group-randomized controlled trial. J Sport Exerc Psychol. 2015;37: 575–591. 10.1123/jsep.2015-0069 26866766

[28] Pesce C, Crova C, Marchetti R, Struzzolino I, Masci I, Vannozzi G, et al Searching for cognitively optimal challenge point in physical activity for children with typical and atypical motor development. Ment Health Phys Act. 2013;6: 172–180.

[29] Kahneman D, Tursky B, Shapiro D, Crider A. Pupillary, heart rate, and skin resistance changes during a mental task. J Exp Psychol. 1969;79: 164–167. 5785627 10.1037/h0026952

[30] Mukherjee S, Yadav R, Yung I, Zajdel DP, Oken BS. Sensitivity to mental effort and test-retest reliability of heart rate variability measures in healthy seniors. Clin Neurophysiol. 2011;122: 2059–2066. 10.1016/j.clinph.2011.02.032 21459665 PMC3132243

[31] Luft CD, Takase E, Darby D. Heart rate variability and cognitive function: effects of physical effort. Biol Psychol. 2009;82: 164–168. 10.1016/j.biopsycho.2009.07.007 19632295

[32] Kimhy D, Crowley O V, McKinley PS, Burg MM, Lachman ME, Tun PA, et al The association of cardiac vagal control and executive functioning-findings from the MIDUS study. J Psychiatr Res. 2013;47: 628–635. 10.1016/j.jpsychires.2013.01.018 23434176 PMC3594003

[33] O’Loughlin EK, Dugas EN, Sabiston CM, O’Loughlin JL. Prevalence and correlates of exergaming in youth. Pediatrics. 2012;130: 806–814. 10.1542/peds.2012-0391 23027171

[34] Moodithaya S, Avadhany ST. Gender differences in age-related changes in cardiac autonomic nervous function. J Aging Res. 2012;2012. 10.1155/2012/679345 PMC3236491 22187649

[35] Staiano AE, Abraham AA, Calvert SL. Competitive versus cooperative exergame play for African American adolescents’ executive function skills: Short-term effects in a long-term training intervention. Dev Psychol. 2012;48: 337–342. 10.1037/a0026938 22369339 PMC4097099

[36] Flynn RM, Richert RA, Staiano AE, Wartella E, Calvert SL. Effects of exergame play on EF in children and adolescents at a summer camp for low income youth. J Educ Develop Psychol. 2014;4(1):209–225. 10.5539/jedp.v4n1p209 25328562 PMC4196243

[37] Faul F, Erdfelder E, Lang A-G, Buchner A. G* Power 3: A flexible statistical power analysis program for the social, behavioral, and biomedical sciences. Behav Res Methods. 2007;39: 175–191. 17695343 10.3758/bf03193146

[38] Watzlawik M. Die Erfassung des Pubertätsstatus anhand der Pubertal Development Scale. [Measuring pubertal status with the pubertal development scale]. Diagnostica. 2009;55(1):55–65.

[39] Boudreau B, Poulin C. An examination of the validity of the Family Affluence Scale II (FAS II) in a general adolescent population of Canada. Soc Indic Res. 2008;94: 29–42.

[40] Fuchs R, Klaperski S, Gerber M, Seelig H. Messung der Bewegungs- und Sportaktivität mit dem BSA-Fragebogen. [Measurement of physical activity and sport activity with the BSA questionnaire]. Z Gesundheitspsychol. 2015;23(2):60–76.

[41] Borg GA. Psychophysical bases of perceived exertion. Med Sci Sport Exerc. 1982;14: 377–381. Available: http://www.ncbi.nlm.nih.gov/pubmed/7154893 7154893

[42] Schmidt M, Benzing V, Kamer M. Classroom-Based Physical Activity Breaks and Children’s Attention: Cognitive Engagement Works! Front Psychol. 2016;7: 1–13. 10.3389/fpsyg.2016.00001 27757088 PMC5047899

[43] Bradley MM, Lang PJ. Measuring emotion: the self-assessment manikin and the semantic differential. J Behav Ther Exp Psychiatry. 1994;25(1):49–59. Available: http://www.ncbi.nlm.nih.gov/pubmed/7962581 7962581 10.1016/0005-7916(94)90063-9

[44] Delis DC, Kaplan E, Kramer JH. Delis-Kaplan executive function system (D-KEFS). Psychological Corporation; 2001: 78–90.

[45] Baggetta P, Alexander PA. Conceptualization and operationalization of executive function. Mind, Brain, Educ. 2016;10: 10–33.

[46] Homack S, Lee D, Riccio CA. Test Review: Delis-Kaplan Executive Function System. J Clin Exp Neuropsychol. 2005;27: 599–609. 10.1080/13803390490918444 16019636

[47] Task Force of the European Society of Cardiology and the North American Society of Pacing and Electrophysiology. Heart rate variability standards of measurement, physiological interpretation, and clinical use. Eur Heart J. 1996;17:354–381. 8737210

[48] Tarvainen MP, Niskanen JP, Lipponen JA, Ranta-Aho PO, Karjalainen PA. Kubios HRV-heart rate variability analysis software. Comput Methods Programs Biomed. 2014;113: 210–220. 10.1016/j.cmpb.2013.07.024 24054542

[49] Vickers AJ, Altman DG. Statistics Notes: Analysing controlled trials with baseline and follow up measurements. BMJ. 2001;323: 1123–1124. 11701584 10.1136/bmj.323.7321.1123 PMC1121605

[50] Van Breukelen GJP. ANCOVA versus change from baseline had more power in randomized studies and more bias in nonrandomized studies. J Clin Epidemiol. 2006;59: 920–925. 10.1016/j.jclinepi.2006.02.007 16895814

[51] Chang YK, Chu CH, Wang CC, Wang YC, Song TF, Tsai CL, et al Dose-response relation between exercise duration and cognition. Med Sci Sport Exerc. 2015;47: 159–165. 10.1249/MSS.0000000000000383 24870572

[52] Chang YK, Labban JD, Gapin JI, Etnier JL. The effects of acute exercise on cognitive performance: A meta-analysis. Brain Res. 2012;1453: 87–101. 10.1016/j.brainres.2012.02.068 22480735

[53] Gale CR, Batty GD, Tynelius P, Deary IJ, Rasmussen F. Intelligence in early adulthood and subsequent hospitalization for mental disorders. Epidemiology. 2010;21: 70–77. 10.1097/EDE.0b013e3181c17da8 19907333 PMC4170757

[54] Faulkner MS, Hathaway D, Tolley B. Cardiovascular autonomic function in healthy adolescents. Heart Lung. 2003;32: 10–22. 10.1067/mhl.2003.6 12571544

[55] Budde H, Pietrassyk-Kendziorra S, Bohm S, Voelcker-Rehage C. Hormonal responses to physical and cognitive stress in a school setting. Neurosci Lett. 2010;474: 131–134. 10.1016/j.neulet.2010.03.015 20226843

[56] Budde H, Windisch C, Kudielka BM, Voelcker-Rehage C. Saliva cortisol in school children after acute physical exercise. Neurosci Lett. 2010;483: 16–19. 10.1016/j.neulet.2010.07.036 20654695

[57] Wegner M, Koedijker JM, Budde H. The effect of acute exercise and psychosocial stress on fine motor skills and testosterone concentration in the saliva of high school students. PLoS One. 2014;9: e92953 10.1371/journal.pone.0092953 24664108 PMC3963958

[58] Pendleton DM, Sakalik ML, Moore ML, Tomporowski PD. Mental engagement during cognitive and psychomotor tasks: Effects of task type, processing demands, and practice. Int J Psychophysiol. 2016. 10.1016/j.ijpsycho.2016.08.012 27585951

[59] Hogan M, Kiefer M, Kubesch S, Collins P, Kilmartin L, Brosnan M. The interactive effects of physical fitness and acute aerobic exercise on electrophysiological coherence and cognitive performance in adolescents. Exp Brain Res. 2013;229: 85–96. 10.1007/s00221-013-3595-0 23743717

[60] Hillman C., Kamijo K., & Scudder M. A review of chronic and acute physical activity participation on neuroelectric measures of brain health and cognition during childhood. Prev Med. 2011;52: 1–15. 21281669 10.1016/j.ypmed.2011.01.024 PMC3094734

[61] Hillman CH, Pontifex MB, Raine LB, Castelli DM, Hall EE, Kramer AF. The effect of acute treadmill walking on cognitive control and academic achievement in preadolescent children. Neuroscience. 2009;159: 1044–1054. 10.1016/j.neuroscience.2009.01.057 19356688 PMC2667807

[62] Suchy Y, Kraybill ML, Gidley Larson JC. Understanding design fluency: motor and executive contributions. J Int Neuropsychol Soc. 2010;16: 26–37. 10.1017/S1355617709990804 19796444

