# Supplementary material for: Acute Cognitively Engaging Exergame-Based Physical Activity Enhances Executive Functions in Adolescents
Source: PLoS One. 2016 Dec 28;11(12):e0167501. doi: 10.1371/journal.pone.0167501 (PMC5193332; doi:10.1371/journal.pone.0167501)
Supplement: S1 Table — (DOCX) [file pone.0167501.s001.docx]

**S1 Table. Overview of studies examining acute effects of physical exercises on cognitive performance, differing in the level of cognitive engagement.**

| **Authors** | **Sample size** | **Modality** | **Intensity** (heart rate) | **Duration** | **Age** | **Assessments** | **Statistics** | **Results** |
| --- | --- | --- | --- | --- | --- | --- | --- | --- |
| Budde et al., 2008 [1] | 99 | Normal sport lesson (NSL) vs. Coordinative Exercise (CE) | NSL: *M* = 121.96 (*SD* = 27.06); CE: *M* = 122.30 (*SD* = 21.91) | App. 10 min. each condition | 13-16 years | Inhibition (d2) | ANOVA | NSL < CE |
| Pesce et al., 2009  [2] | 60 | Circuit training (CT) vs. Team Games (TG) | CT: *M* = 146 (*SD* = 19); TG: *M* = 137 (*SD* = 18) | App. 42 min. each condition | 11-12 years | Free recall memory task | MANOVA | CT < TG |
| Schmidt et al., 2015 [3] | 90 | Control (C) vs. Cognitively demanding Physical Education lesson (CPE) | Not considered | App. 45 min. each condition | 11-12 years | Inhibition (d2-R) | ANOVA | C < CPE (after 90 minutes) |
| Best, 2012 [4] | 33 | Low Physical Activity (PA), low Cognitive Engagement (CE) vs. low PA, high CE vs. high PA, low CE vs. high PA, high CE | Low PA, low CE: *M* = 93.1 (*SD* = 2.5); Low PA, High CE: *M* = 94.2 (*SD* = 2.2); High PA, low CE: *M* = 154.8 (*SD* = 3.8); High PA, high CE: *M* = 157.9 (*SD* = 2.9) | App. 23 min. each condition | 6-10 years | Inhibition (Flanker task) | Mixed-model ANOVA | Low PA < high PA; high PA = high PA, high CE |
| Jäger et al., 2015 [5] | 217 | Control (C) vs. Cognitive Games (CG) vs. Aerobic Exercise (AE) vs. Physical Games (PG) | C: *M* = 81.93 (*SD* = 10.13); CG: *M* = 94.52 (*SD* = 8.71); AE: *M* = 150.76 (*SD* = 15.14); PG: *M* = 147.79 (*SD* = 17.62) | App. 20 min each condition | 10-12 years | Updating (non-spatial n-back task); Inhibition (Flanker task); Shifting (Flanker task) | ANOVA | No significant differences between groups |
| Gallotta et al., 2012 [6] | 138 | Physical Exertion (PE) vs. Cognitive Exertion (CE) vs. Cognitive and Physical Exertion (CPE) | PE: *M* = 146.56 (*SD* = 14.09); CPE: *M* = 147.25 (*SD* = 15.50) | App. 50 min each condition (Physical conditions: 15 min warm up; 30 min moderate to vigorous PA; 5 min stretching) | 8-11 years | Inhibition (d2) | ANOVA | CPE < CE < PE |
| Gallotta et al., 2015 [7] | 116 | Physical Exertion (PE) vs. Cognitive Exertion (CE) vs. Cognitive and Physical Exertion (CPE) | PE: *M* = 146.56 (*SD* = 14.09); CPE: *M* = 147.25 (*SD* = 15.50) | App. 50 min each condition (Physical conditions: 15 min warm up; 30 min moderate to vigorous PA; 5 min stretching) | 8-11 years | Inhibition (d2) | Repeated measures ANOVA | CPE < CE < PE |

**References**

1. Budde H, Voelcker-Rehage C, Pietraßyk-Kendziorra S, Ribeiro P, Tidow G. Acute coordinative exercise improves attentional performance in adolescents. *Neurosci Lett*. 2008;441(2):219-23.
2. Pesce C, Crova C, Cereatti L, Casella R, Bellucci M. Physical activity and mental performance in preadolescents: Effects of acute exercise on free-recall memory. *Ment Health Phys Act*. 2009;2(1):16-22.
3. Schmidt M, Egger F, Conzelmann A. Delayed positive effects of an acute bout of coordinative exercise on children's attention. *Percept Mot Skills*. 2015; 121(2), 431-446.
4. Best JR. Exergaming immediately enhances children's executive function. *Dev Psychol*. 2012;48(5):1501-10.
5. Jäger K, Schmidt M, Conzelmann A, Roebers CM. The effects of qualitatively different acute physical activity interventions in real-world settings on executive functions in preadolescent children. *Mental Health and Physical Activity*. 2015;9:1-9.
6. Gallotta MC, Guidetti L, Franciosi E, Emerenziani GP, Bonavolonta V, Baldari C. Effects of varying type of exertion on children's attention capacity. *Med Sci Sports Exerc*. 2012;44(3):550-5.
7. Gallotta MC, Emerenziani GP, Franciosi E, Meucci M, Guidetti L, Baldari C. Acute physical activity and delayed attention in primary school students. *Scand J Med Sci Sports*. 2015;25(3):e331-8.
